# Paxillin participates in the sphingosylphosphorylcholine-induced abnormal contraction of vascular smooth muscle by regulating Rho-kinase activation

**DOI:** 10.1186/s12964-023-01404-w

**Published:** 2024-01-22

**Authors:** Ying Zhang, Nan Li, Sei Kobayashi

**Affiliations:** Department of Molecular and Cellular Physiology, Graduate School of Medicine, Yamaguchi University, 1-1-1 Minami-Kogushi, Ube, Yamaguchi 755-8505 Japan

**Keywords:** Sphingosylphosphorylcholine, Vascular smooth muscle cell, Vascular contraction, Paxillin, Fyn, Rho-kinase

## Abstract

**Background:**

The Ca^2+^-independent contraction of vascular smooth muscle is a leading cause of cardiovascular and cerebrovascular spasms. In the previous study, we demonstrated the involvement of Src family protein tyrosine kinase Fyn and Rho-kinase in the sphingosylphosphorylcholine (SPC)-induced abnormal and Ca^2+^-independent contraction of vascular smooth muscle, but the specific mechanism has not been completely clarified.

**Methods:**

Paxillin knockdown human coronary artery smooth muscle cells (CASMCs) and smooth muscle-specific paxillin knockout mice were generated by using paxillin shRNA and the tamoxifen-inducible Cre-LoxP system, respectively. CASMCs contraction was observed by time-lapse recording. The vessel contractility was measured by using a myography assay. Fyn, Rho-kinase, and myosin light chain activation were assessed by immunoprecipitation and western blotting. The paxillin expression and actin stress fibers were visualized by histological analysis and immunofluorescent staining.

**Results:**

The SPC-induced abnormal contraction was inhibited in paxillin knockdown CASMCs and arteries of paxillin knockout mice, indicating that paxillin is involved in this abnormal contraction. Further study showed that paxillin knockdown inhibited the SPC-induced Rho-kinase activation without affecting Fyn activation. In addition, paxillin knockdown significantly inhibited the SPC-induced actin stress fiber formation and myosin light chain phosphorylation. These results suggest that paxillin, as an upstream molecule of Rho-kinase, is involved in the SPC-induced abnormal contraction of vascular smooth muscle.

**Conclusions:**

The present study demonstrated that paxillin participates in the SPC-induced abnormal vascular smooth muscle contraction by regulating Rho-kinase activation.

Video Abstract

**Supplementary Information:**

The online version contains supplementary material available at 10.1186/s12964-023-01404-w.

## Background

Myocardial infarction and cerebral infarction caused by coronary artery spasm and cerebrovascular spasm are considered to be among the main causes of morbidity and mortality worldwide. This vasospasm is an abnormal and long-lasting contraction of vascular smooth muscle (VSM), which leads to narrowing of blood vessels and reduces blood flow to tissues and organs, eventually resulting in ischemia [1–4]. Many studies have demonstrated that this abnormal contraction is characterized by the Ca^2+^-independent contraction or Ca^2+^-sensitization [5–7], which differs from the normal vascular tone that is characterized by the Ca^2+^-dependent contraction mediated through Ca^2+^-calmodulin (CaM)-myosin light chain kinase (MLCK) pathway [8–10]. Our previous study has shown that sphingosylphosphorylcholine (SPC), a bioactive sphingolipid produced by the hydrolysis of the membrane sphingolipid, triggers the Ca^2+^-independent contraction of VSM [6, 7]. SPC concentration in the cerebrospinal fluid significantly increases after subarachnoid hemorrhage and is considered to be the main cause of cerebrovascular spasm [11], acting as a spasmogen.

Fyn tyrosine kinase, a member of the Src family protein tyrosine kinases, and Rho-kinase have been shown to be involved in the SPC-induced abnormal contraction of VSM [5, 7]. In our previous study, we proposed that Fyn translocation from the cytosol to the cell membrane is involved in the SPC-induced contraction of VSM. Additionally, inhibition of Fyn translocation by EPA effectively inhibits the SPC-induced contraction [5]. The active Fyn regulates the SPC-induced actin stress fiber formation and constitutively active Fyn (CA-Fyn) is located at the ends of actin stress fibers anchored at focal adhesion [12, 13]. Accumulating evidence has demonstrated that Rho-kinase mediates coronary artery spasm and cerebrovascular spasm [14–20], including the SPC-induced contraction of VSM [5–7, 21]. Dominant negative Rho-kinase and Rho-kinase inhibiter Y27632 abolish the SPC-induced contraction [6, 7]. Fasudil, another Rho-kinase inhibitor, has been shown to effectively prevent coronary artery spasm and cerebral vasospasm [16, 18]. Translocation of Rho-kinase from the cytosol to the cell membrane in VSM cells plays an essential role in the SPC-induced contraction [6]. Although Rho-kinase and Fyn have been implicated in the regulation of the SPC-induced contraction of VSM, the precise mechanism(s) underlying this abnormal contraction of VSM remains largely unclear.

Focal adhesions are large and dynamic protein complexes containing paxillin and focal adhesion kinase (FAK), which connect the extracellular matrix to cytoskeletal microfilaments and play a fundamental role in cell migration. Recent research has shown that focal adhesions play a critical role in muscle contraction by acting as sites for transmitting the contractile force generated by the actin-myosin stress fiber network to the rigid extracellular matrix [22–24]. FAK activation facilitates smooth muscle contraction in the gastric fundus and depletion of FAK inhibits contractile activation of smooth muscle [25]. Paxillin plays an important role in cytoskeletal reorganization by recruiting various signaling molecules, including FAK [26–29]. Paxillin, as a substrate for FAK, regulates the Ca^2+^-dependent contraction of tracheal smooth muscle [30]. Loss of paxillin in zebrafish results in decreased cardiac contractility, leading to severe heart failure [31]. Recently, we have discovered that paxillin is a binding partner of activated Fyn [32]. Based on these findings, we hypothesized that paxillin may play a role in the SPC-induced abnormal contraction of VSM.

In this study, we aimed to investigate the involvement of paxillin in the SPC-induced abnormal contraction of VSM. We utilized paxillin knockdown human coronary artery smooth muscle cells (CASMCs) and smooth muscle-specific paxillin knockout mice to examine their SPC-induced contractions. Furthermore, we examined the mechanism(s) by which paxillin regulates the SPC-induced contraction of VSM. Our findings revealed a previously unknown role for paxillin in the SPC-induced abnormal contraction of VSM, highlighting its potential as a therapeutic target for reducing cardiovascular and cerebrovascular diseases associated with abnormal VSM contraction.

## Methods and materials

### Reagents and antibodies

Sphingosylphosphorylcholine (SPC) was purchased from Biomol (Plymouth Meeting, PA, USA). Tamoxifen was purchased from Funakoshi (Tokyo, Japan). Corn oil was purchased from Sigma Aldrich (St. Louis, MO, USA).

The following antibodies were used: mouse monoclonal anti-paxillin antibody (Cat#610620, BD Biosciences, San Jose, CA, USA), anti-paxillin polyclonal antibody (H85, Santa Cruz, Dallas, CA, USA), anti-GAPDH monoclonal antibody (Wako, Osaka, Japan), anti-phospho-MYPT1 (T850) with phosphorylation site corresponding to T853 in human MYPT1 (Milipore, Burlington, CA, USA), anti-MYPT1 antibody (H130, Santa Cruz, Dallas, CA, USA), anti-phospho-myosin light chain 2 (S19) monoclonal antibody (Cell Signaling, Danvers, MA, USA), anti-myosin light chain (20 kDa) monoclonal antibody (Sigma Aldrich, St. Louis, MO, USA), anti-α-smooth muscle actin antibody (Thermo Fisher, Waltham, MA, USA), anti-mouse Alexa Fluor 488 (Thermo Fisher, Waltham, MA, USA), and anti-rabbit Alexa Fluor 546 (Thermo Fisher,Waltham, MA, USA). Secondary HRP-labeled antibodies (anti-mouse and anti-rabbit) were purchased from Promega (Madison, WI, USA).

### Cell culture

Human coronary artery smooth muscle cells (CASMCs) were cultured in HuMedia SG2 (Kurabo, Osaka, Japan) supplemented with 5% fetal bovine serum (FBS), 0.5 ng/ml human epidermal growth factor (hEGF), 2 ng/ml human fibroblast growth factor-B (hFGF-B), 5 µg/ml insulin, 50 µg/ml gentamycin, and 50 ng/ml amphotericin B. Cells were maintained at 37 °C in a humidified atmosphere of 5% CO_2_ and 95% air. Human CASMCs at passage numbers < 10 splitting cycles were used for experiments.

To generate paxillin-downregulated cells, we infected human CASMCs with paxillin shRNA lentiviral particles (sc-29439-V, Santa Cruz, Dallas, CA, USA) following the manufacturer’s instructions. Prior to infection, we optimized the infection conditions using copGFP control lentiviral particles (sc-108084, Santa Cruz, Dallas, CA, USA) as previously reported [32]. Using the optimal condition, we successfully generated a paxillin-downregulated cell line. In brief, cells were seeded in a 12-well plate and were infected with paxillin shRNA or control shRNA (sc-108080, Santa Cruz, Dallas, CA, USA) lentiviral particles mixed with complete medium and Polybrene (sc-134220, Santa Cruz, Dallas, CA, USA) when they reached approximately 50% confluency. After infection, stable clones were selected using puromycin dihydrochloride (sc-108071, Santa Cruz, Dallas, CA, USA) at the concentration of 2 µg/mL.

### Immunoprecipitation and western blotting assay

Serum-starved cells were treated with SPC (30 µM) for the indicated times. The cells were washed with PBS and lysed using an immunoprecipitation assay buffer (50 mM Tris, pH 7.4, 150 mM NaCl, 10 mM NaF, 0.2 mM Na_3_VO_4_, 0.1% Triton X-100, 0.5% NP-40, 1 mM phenylmethylsulphonyl fluoride [PMSF], 1 µg/mL of leupeptin, and 1 µg/mL of aprotinin). Protein concentrations were determined using a protein assay kit (Bio-Rad, Hercules, CA, USA) with bovine serum albumin as the standard. The cell lysates containing equal amounts of proteins were centrifuged at 4 °C for 10 min at 10,000×*g* and the supernatants were precleared by incubation with protein A/G agarose beads (Santa Cruz, Dallas, CA, USA) at 4 °C for 1 h, followed by centrifugation at 4 °C for 5 min at 10,000×*g*. The supernatants were then incubated with 1 µg of Fyn antibody and protein A/G agarose beads overnight at 4 °C. After washing the beads four times with ice-cold immunoprecipitation assay buffer, the bound proteins were eluted with 2 × SDS-PAGE sample buffer (2% SDS, 20 mM DTT, 20% glycerol, 20 mM Tris pH 6.8, and 0.1% bromophenol blue). The samples were separated using 10% SDS-PAGE and transferred onto Amersham Hybond-P PVDF membrane (GE Healthcare Life Sciences, Baie d’Urfé, QC, Canada), followed by the blocking of the membrane with 5% non-fat milk in TBS-T for 60 min at 25 °C. The membranes were incubated with primary antibodies for 1 h at 25 °C or overnight at 4 °C, followed by their incubation with peroxidase-conjugated secondary antibodies for 1 h at 25 °C. Immunoreactive bands were detected using SuperSignal West Pico chemiluminescent substrate kit (Thermo Fisher Scientific, Waltham, MA, USA) and digitally quantified using Bio-Rad ChemiDoc XRS-J detection system.

Tissue samples were collected from mice as described in our previous report [33]. Briefly, the smooth muscle layer of the aorta was isolated after the removal of adipose tissue and adventitia under an optical microscope. Subsequently, the layer was sectioned into strips and subjected to treatment either in the absence (vehicle control) or in the presence of SPC (10 µM for 15 min at 37 °C). The tissues were then promptly frozen in a solution of 10% trichloroacetic acid (TCA) and 10 mM DTT in acetone, which had been pre-chilled on ice, and subsequently rinsed twice with cold 10 mM DTT/acetone. The tissues were then placed in liquid nitrogen and pulverized using the SK-Mill Freeze-Crush Apparatus. Protein was extracted from the freeze-dried tissues by adding 100 µL of RIPA buffer (Wako, Osaka, Japan) along with a protease inhibitor cocktail (Sigma-Aldrich, St. Louis, MO, CA), and the samples were heated at 95 °C for 5 min for western blot analysis.

### Time-lapse recording of VSM cell contraction

Human CASMCs infected with lentiviral control shRNA or paxillin shRNA were grown in 35-mm glass-based dishes (Iwaki, Osaka, Japan). When cell confluence reached 90–100%, FBS and growth factor-free HuMedia SB2 (Kurabo, Osaka, Japan) medium was changed to induce hypercontractile type of CASMCs following previously established methods [33]. After treatment with HuMedia SB2 for 48 h, 30 µM SPC was added to the medium and time-lapse recording of VSM cell contraction was performed under a fluorescent microscope (Keyence Biorevo BZ-9000, Osaka, Japan). Cell images were recorded for 30 min every 30 s.

### Animals

C57BL/6 mice at 6–18 weeks old were used for experiments. SMMHC-CreER^T2^ transgenic mice were purchased from The Jackson Laboratory (RRID:IMSR_JAX:019079). The offspring homozygous mice were produced in the Institute of Life Science and Medicine of Yamaguchi University. Mice were kept with a 12 h light-dark border circulation system (25℃) under a pathogen-free condition with free access to food and water. All mice utilized in this study and all experiments were conducted in accordance with the Animal Care and Use Committee of the Model Animal Research Center at Yamaguchi University. Animal welfare and experimental programs strictly comply with the guidelines for the care and use of laboratory animals.

### Generation of smooth muscle tissue-specific paxillin knockout mice (paxillin SMKO mice)

Major experimental tasks (design and construction of targeting vector, establishment of targeted ES cells, generation of the chimera and F1 mice, and removal of the neo cassette) to generate the paxillin SMKO mice were performed by Unitech, Co. (Kashiwa, Japan). Briefly, bacterial artificial chromosome (BAC)-retrieval methods were used for constructing the targeting vector (Additional file 1: Figure S1). Exons 2–5, which encode paxillin, were flanked by 2 loxP sites and an *frt-Neo-frt* cassette as a positive selection marker. The “FRT-Neo-FRT-loxP” sequence was inserted upstream of Exon 2, and the loxP sequence was inserted downstream of Exon 5. Therefore, when the region containing Exons 2 to 5 flanked by loxP sequences was removed by the Cre-loxP system, the region after Exon 2 of the target gene was knocked out by frameshifting.

To specifically ablate the expression of paxillin in smooth muscle cells, we generated mice with two floxed paxillin alleles and the SMMHC-CreER^T2^ transgene by crossing paxillin floxed mice with SMMHC-CreER^T2^ transgenic mice, as shown in Additional file 2: Figure S2A. DNA isolated from mice tail tissues were genotyped by PCR for LoxP^+/+^ sites and the presence of the Cre-recombinase using specific primers (For LoxP^+/+^, forward primer sequence: 5′-AAACCTTTCTTCATAAATTGGAAGG-3′, the revers primer sequence: 5′-GGAAGGTATAGATGTGTATCAGCAC-3′; For Cre, the forward primer sequence: 5′-TGACCCCATCTCTTCACTCC-3′, the reverse primer sequence: 5′-AGTCCCTCACATCCTCAGGTT-3′). A representative genotyping result showed Lox P^+/+^ sites (350 bp), Cre control (287 bp) and wild type (190 bp) in Additional file 2: Figure S2B, S2C, and S2D. Male mice received intraperitoneal injections of tamoxifen at a dose of 1 mg tamoxifen solution in 100 µl corn oil for five consecutive days [34, 35]. Tamoxifen (T5648, Sigma-Aldrich, St. Louis, MO, USA) was dissolved in corn oil (42 °C) at a concentration of 10 mg/mL and incubated at 37 °C for 1 h before use. After four weeks of tamoxifen injections, the mice were used for experiments. Only male mice were used in this study, as the SMMHC-CreER^T2^ transgene is located on the Y-chromosome.

### Myography assay for vessel contractility

Male mice injected with corn oil (control mice) and tamoxifen (paxillin SMKO mice) were sacrificed by cervical dislocation, and thoracic aorta was excised. Connective tissue and fat adjacent to the aorta were carefully removed without causing any damage to the endothelium. The aorta was cut into several 2 mm rings and suspended in a multi-wire myograph system (DMT610M, Lab Tech, Japan) and bathed in Krebs solution (123 mM NaCl, 4.7 mM KCl, 15.5 mM NaHCO_3_, 1.2 mM KH_2_PO_4_, 1.2 mM MgCl_2_, 1.25 mM CaCl_2_, and 11.5 mM D-glucose) gassed with 95% O_2_/5% CO_2_. After equilibration for 30 min at 37℃, the rings were contracted with 80 mM K^+^-physiological salt solution (48.9 mM NaCl, 78.8 mM KCl, 15.5 mM NaHCO_3_, 1.2 mM KH_2_PO_4_, 1.2 mM MgCl_2_, 1.25 mM CaCl_2_, and 11.5 mM D-glucose) and washed three times with Krebs solution. After recording steady responses to repeated applications of 80 mM K^+^-depolarization, endothelium inhibitor L-NAME was added to inhibit eNOS for 15 min, and then 80 mM K^+^-depolarization was used to induce contraction again. After re-adding L-NAME for another 15 min, VSM contraction was induced using different concentrations of SPC (1, 3, 10, and 30 µM) or noradrenaline (0.001, 0.003, 0.01, 0.03, 0.1, 0.3, 1, 3, 10, and 30 µM).

### Histological analysis and immunofluorescent staining

For the histological analysis, tissues were fixed overnight using 4% paraformaldehyde and then embedded in paraffin. Transverse Sect. (5 μm) were stained with hematoxylin/eosin and examined under a microscope (Keyence Biorevo BZ-9000, Osaka, Japan). For the immunofluorescence analysis, sections were blocked with NanoBio blocker solution (Nano Bio Tech Co., Ltd) diluted in PBS for 30 min at 25℃. After washing three times with PBS, primary antibodies and fluorescent secondary antibodies were used. The fluorescent staining was examined with a fluorescent microscope (Keyence Biorevo BZ-9000, Osaka, Japan).

The staining of F-actin was carried out using rhodamine-conjugated phalloidin (1:100, Thermo Fisher Scientific, Waltham, MA, USA) and visualized under a fluorescence microscope (Keyence Biorevo BZ-9000, Osaka, Japan). The formation of actin stress fibers was quantified by counting the number of cells containing a thick linear structure of actin stress fibers.

### Statistical analysis

Data are presented as mean ± SEM. Statistical analysis was performed using GraphPad Prism 8.0. Student’s t-test was used for analyzing data between two groups. Significant differences between groups were considered when *p* < 0.05.

## Results

### Paxillin knockdown inhibits the SPC-induced contraction in human CASMCs

**Fig. 1:**
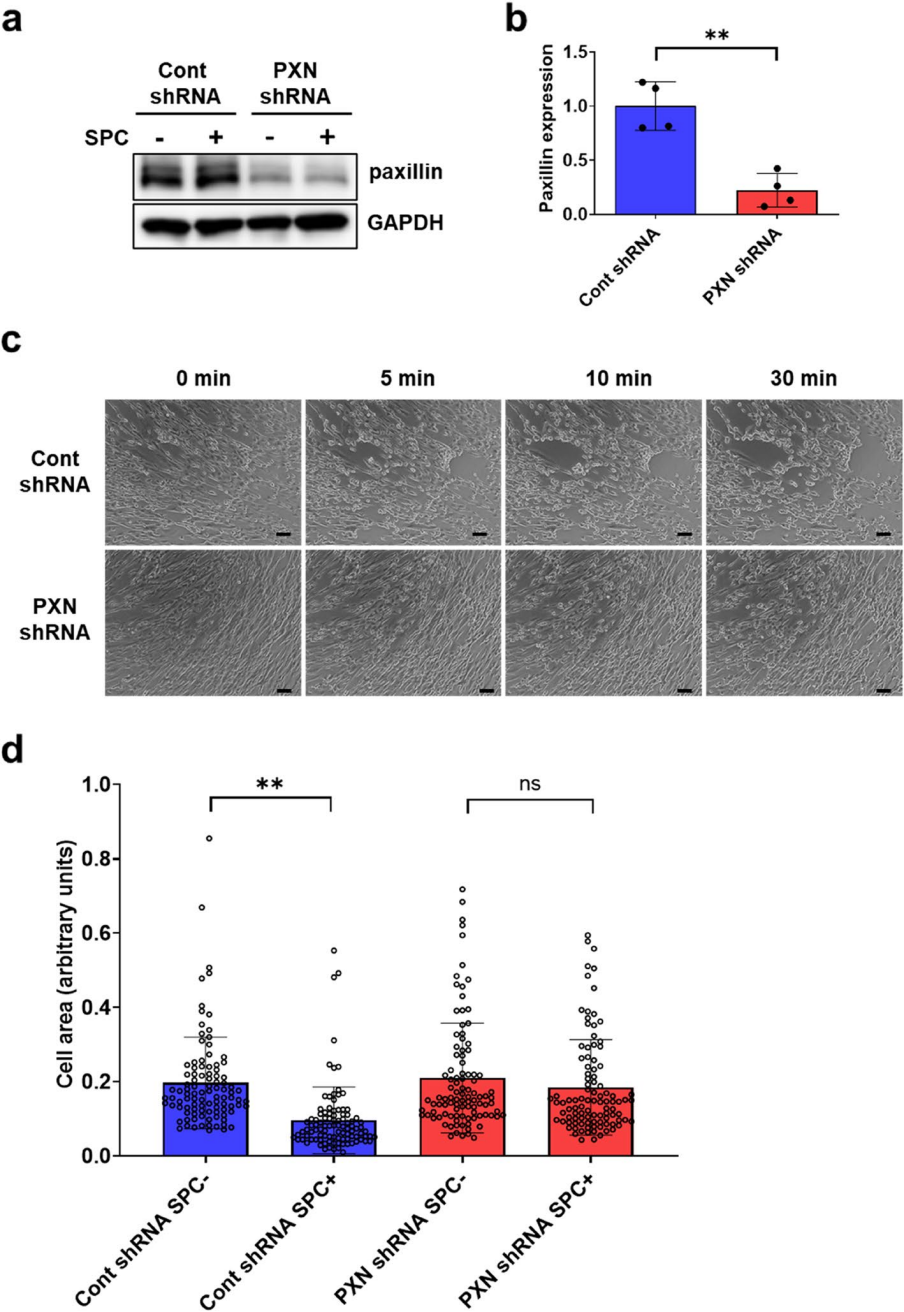
Paxillin knockdown inhibits the SPC-induced contraction in human CASMCs. **a**, Western blot showing paxillin expression in control shRNA or paxillin shRNA lentiviral particles infected human CASMCs in the absence and presence of SPC. **b**, Statistical analysis of paxillin expression in control shRNA and paxillin shRNA lentiviral particles infected human CASMCs (*n* = 4, **p < 0.01). **c**, Representative live cell imaging of cellular contraction at various time points following SPC stimulation (30 µM) in control shRNA and paxillin shRNA lentiviral particles infected human CASMCs. Scale bar = 100 µm. **d**, Statistical analysis of the cell area of control shRNA and paxillin shRNA lentiviral particles infected human CASMCs after SPC stimulation (30 µM) for 30 min. Three independent experiments were performed, and 100 cells were measured for the cell area using ImageJ. **p < 0.01; ns, no significant

To investigate the role of paxillin in the SPC-induced contraction, we first constructed paxillin knockdown human CASMCs using paxillin shRNA lentiviral transduction (Fig. 1a, b). We then recorded the SPC-induced contraction in control and paxillin knockdown human CASMCs through time-lapse observation. As shown in Fig. 1c, SPC induced a noticeable contraction in control CASMCs, with the elongated cells becoming short or round (Additional file 5: Video S1). In contrast, paxillin knockdown human CASMCs showed little change in morphology after stimulation with SPC for 30 min (Additional file 6: Video S2). We quantified cellular area in control and paxillin knockdown CASMCs with and without SPC stimulation. As shown in Fig. 1d, after stimulation with SPC for 30 min, control CASMCs exhibited a significant decrease in cell area compared to the non-stimulated cells. In contrast, the area of paxillin knockdown cells remained relatively unchanged following SPC stimulation for 30 min. These results suggest that paxillin knockdown attenuates the SPC-induced contraction in CASMCs.

### Paxillin deficiency inhibits the SPC-induced contraction in mice

**Fig. 2:**
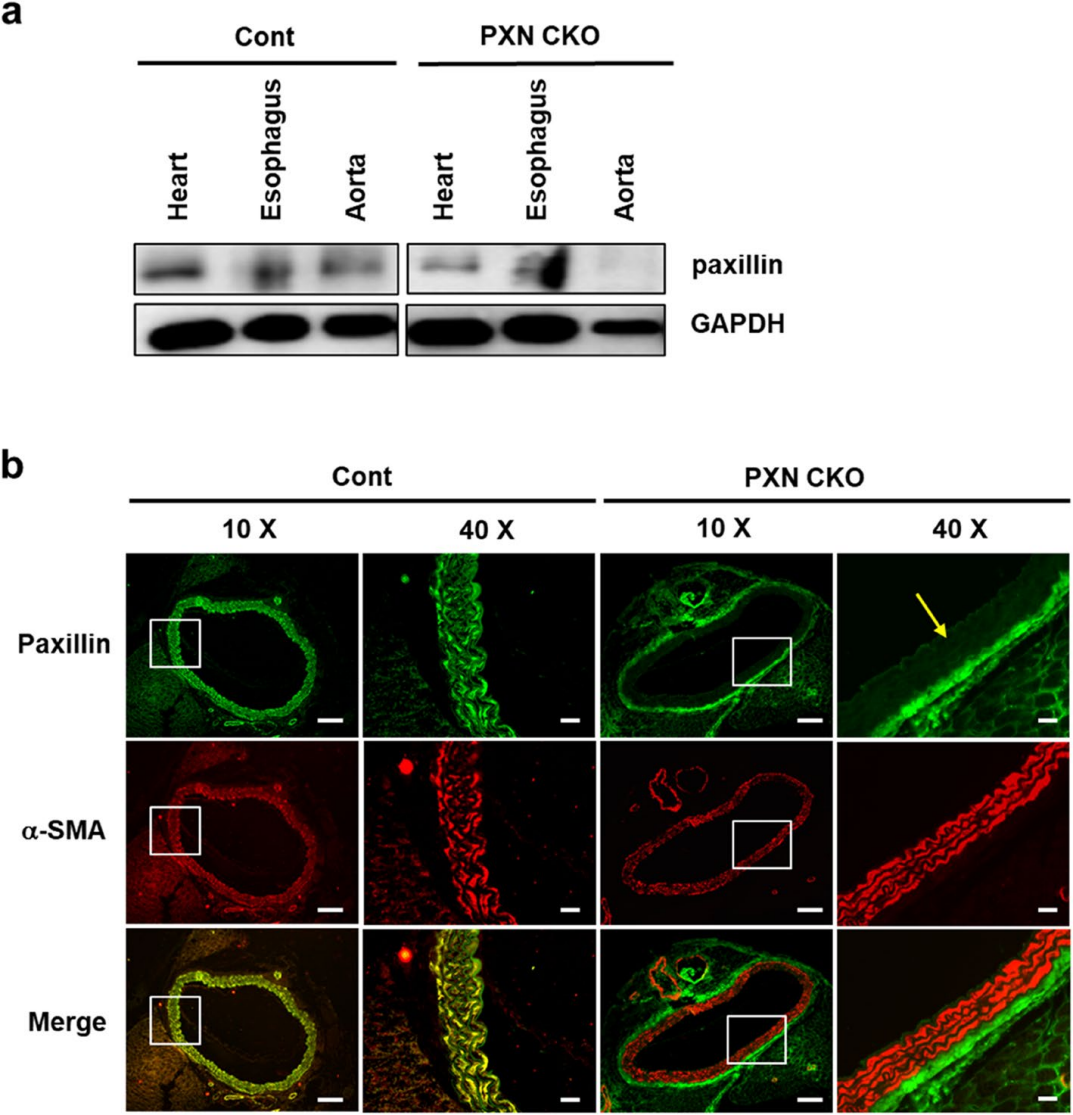
Establishment of paxillin smooth muscle-specific knockout mice. **a**, Representative western blot of paxillin expression in heart muscle, straited muscle layer of esophagus, and the medial smooth muscle layer of thoracic aorta, from control mice (Cont) and paxillin smooth muscle knockout (SMKO) mice (PXN CKO). Note that we removed the endothelial layer and connective tissue, leaving only the medial smooth muscle layer of thoracic aorta. GAPDH is shown as a loading control. **b**, Immunofluorescent staining showing that paxillin is knockout in the medial smooth muscle layer of thoracic aorta of paxillin SMKO mice. α-smooth muscle actin (α-SMA, red) as a smooth muscle-specific marker is detected in both control mice (Cont) and paxillin SMKO mice (PXN CKO). Paxillin (Green) is detected in the medial smooth muscle layer of control mice (Cont) but not in that of paxillin SMKO mice (PXN CKO), as shown in the yellow arrow. Scale Bar = 100 µm in 10× images, Scale Bar = 20 µm in 40× images

To further investigate whether paxillin deficiency in VSM tissue leads to the weakening or loss of the SPC-induced contraction, we generated the paxillin smooth muscle knockout (SMKO) mice using a tamoxifen-inducible Cre-LoxP system. Western blots confirmed the absence of paxillin expression in the medial smooth muscle layer of the thoracic aorta from paxillin SMKO mice (Fig. 2a). Additionally, immunofluorescence staining showed no paxillin expression in the medial smooth muscle layer of the thoracic aorta in paxillin SMKO mice (Fig. 2b).

**Fig. 3:**
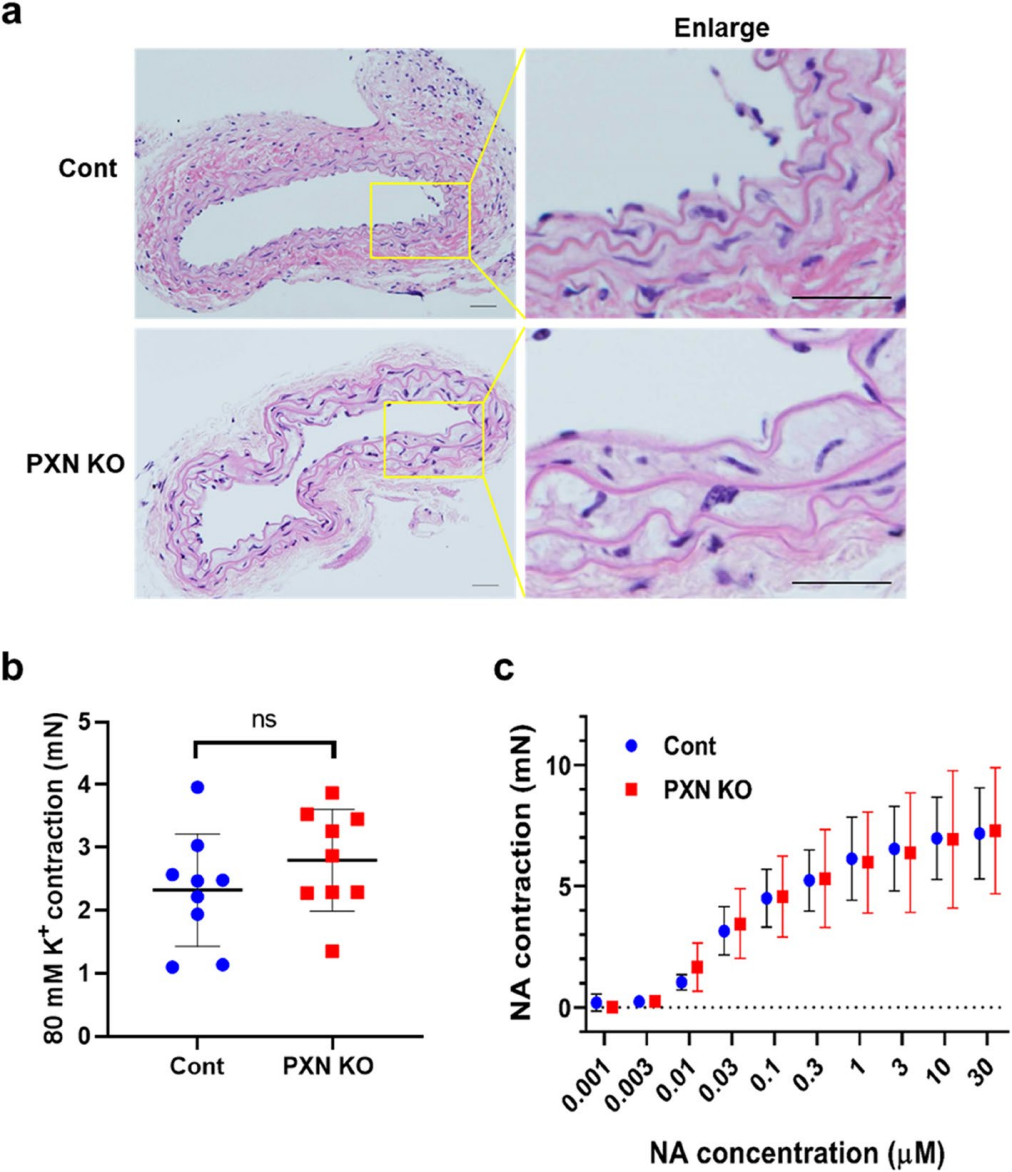
Paxillin knockout has no effect on the physiological structure and function of thoracic aorta. **a**, HE staining of transverse sections of the thoracic aorta in control and paxillin SMKO mice. Scale bar = 200 µm. **b**, 80 mM K^+^ depolarization-induced contraction in thoracic aorta shows no difference between control (*n* = 9) and paxillin SMKO mice (*n* = 9). **c**, Noradrenaline (NA)-induced contraction in thoracic aorta shows a dose-dependent manner from control (*n* = 3) and paxillin SMKO mice (*n* = 3).Cont: control mice; PXN KO: paxillin SMKO mice; ns: no significant

Prior to investigating the SPC-induced contraction of VSM using paxillin SMKO mice, we assessed the structure and function of blood vessels in both control and paxillin SMKO mice. Hematoxylin and eosin (HE) staining revealed no significant difference in the structure of VSM between paxillin SMKO mice and control mice (Fig. 3a). In addition, we examined high K^+^ depolarization-induced and noradrenaline (NA)-induced contractions, which showed no significant differences between control and paxillin SMKO mice (Fig. 3b, c). These results indicate that paxillin deletion in VSM does not affect the structure or contractile function of the artery under normal physiological conditions.

**Fig. 4:**
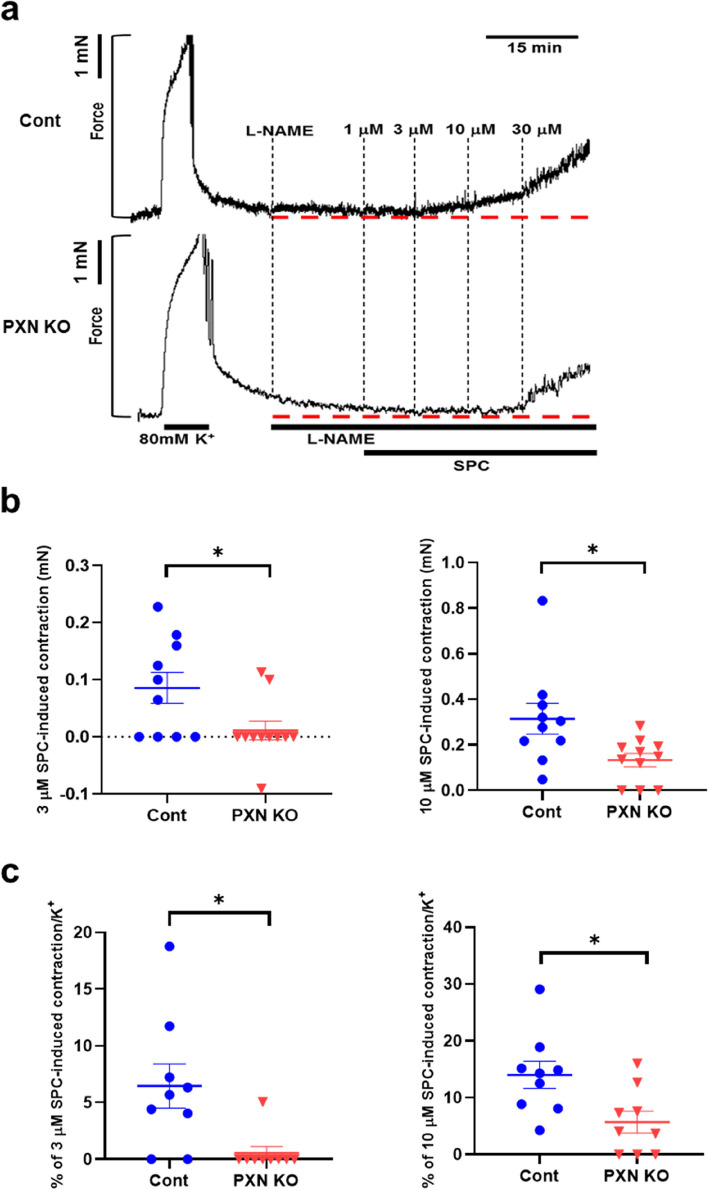
Paxillin knockout attenuates the SPC-induced abnormal contraction of thoracic aorta. **a**, Representative contraction traces are shown for thoracic aorta from control and paxillin SMKO mice after stimulation with SPC at the different concentrations (1, 3, 10, and 30 µM). **b**, The SPC-induced contraction (mN) at the concentration of 3 µM and 10 µM from control (*n* = 10) and paxillin SMKO mice (*n* = 11). *p<0.05. **c**, Ratio of 3 µM and 10 µM SPC-induced contraction compared to 80 mM K^+^ depolarization-induced contraction in control (*n*= 9) and paxillin SMKO mice (*n*= 9). *p<0.05. Cont: control mice; PXN KO: paxillin SMKO mice

We then examined the SPC-induced contraction in the thoracic aorta of control and paxillin SMKO mice. The results, shown in Fig. 4a, depict representative contraction tracings of thoracic aorta strips of control mice and paxillin SMKO mice that were stimulated with varying concentrations of SPC. Figure 4b shows that the SPC-induced contractions (3 µM and 10 µM) were significantly weaker in paxillin SMKO mice compared to the control mice. We also compared the relative value of the SPC-induced contraction to the maximal K^+^-induced contraction. In line with the findings in Fig. 4b, paxillin SMKO mice showed a decrease relative to the control mice when stimulated with SPC at 3 µM and 10 µM concentrations. These results suggest that paxillin plays an important role in the SPC-induced contraction.

### Paxillin deficiency inhibits the SPC-induced Rho-kinase activation, but not Fyn activation

**Fig. 5:**
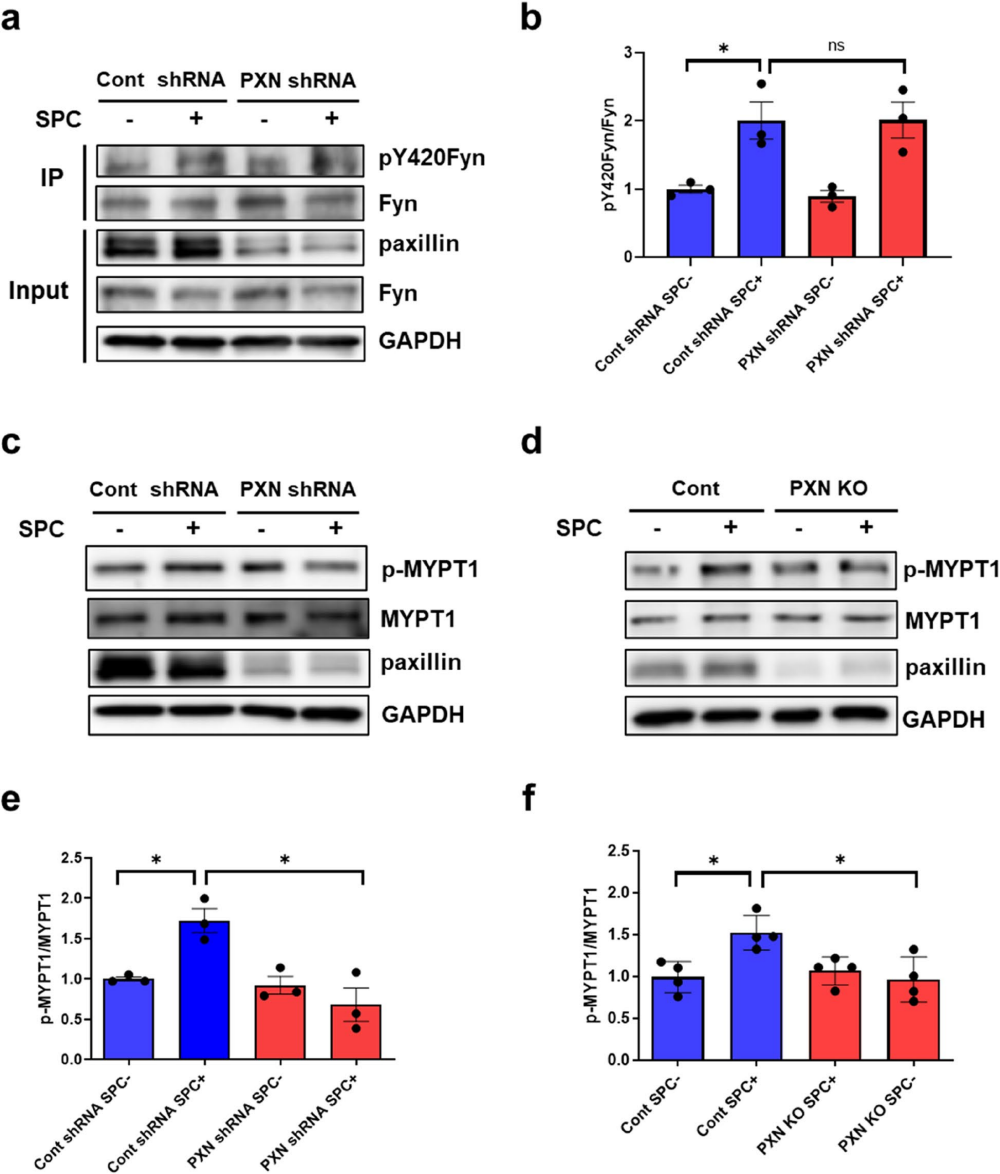
Paxillin knockdown inhibits SPC-induced Rho-kinase activation but not SPC-induced Fyn activation. **a**, Fyn activity in paxillin knockdown human CASMCs in the absence and presence of SPC (30 µM, 5 min), using immunoprecipitation and Western blot. We used Fyn antibody to obtain Fyn and then detected Y420 phosphorylation of Fyn (pY420Fyn) with the Src Y416 phosphorylated antibody that cross-reacts with pY420Fyn. **b**, Statistical analysis of Fyn activation (Ratio of pY420Fyn to total Fyn after Fyn immunoprecipitation) in (a). *n* = 3, *p<0.05, ns: no significant. **c** and **d**, The phosphorylation of MYPT1 at site T853 was analyzed by Western blot in paxillin knockdown human CASMCs (c) and paxillin SMKO mice (d). **e** and **f**, Statistical analysis of Rho-kinase activation (Ratio of phosphorylated MYPT1 at site T853 to total MYPT1) in paxillin knockdown human CASMCs (e,*n* = 3) and paxillin SMKO mice (f,*n* = 4). *p<0.05. Cont: control mice; PXN KO: paxillin SMKO mice

Our recent study demonstrated the association between paxillin and the active Fyn [32], indicating that paxillin may act as a downstream molecule of Fyn. To further investigate this, we examined the activity of Fyn in paxillin knockdown cells. Fyn activity is regulated by Y420 phosphorylation [36]. However, the specific antibody for Fyn Y420 phosphorylation is currently unavailable. Therefore, we utilized an immunoprecipitation method using Fyn antibody to obtain Fyn and then detected Y420 phosphorylation with the use of the Src Y416 phosphorylated antibody that cross-reacts with Y420 phosphorylated Fyn. As shown in Fig. 5a, b, Y420 phosphorylated Fyn increased in both control and paxillin knockdown CASMCs after stimulation of SPC, indicating that paxillin knockdown could not affect the SPC-induced activation of Fyn.

Then we investigated the impact of paxillin deficiency on the SPC-induced Rho-kinase activation. As previously reported, we evaluated the phosphorylation of myosin phosphatase targeting subunit 1 (MYPT1) at T853 [33], which is directly related to Rho-kinase activation. In both control CASMCs and control mice, SPC stimulation significantly increased MYPT1 phosphorylation (Fig. 5c-f). However, in paxillin knockdown CASMCs and paxillin SMKO mice, even in the presence of SPC, MYPT1 phosphorylation did not increase (Fig. 5c-f). These results indicate that paxillin is involved in the SPC-induced Rho-kinase activation and that paxillin serves as an intermediate regulatory molecule linking Fyn and Rho-kinase in SPC-induced contraction.

### Paxillin deficiency attenuates the SPC-induced actin stress fiber formation and myosin light chain (MLC) phosphorylation

**Fig. 6:**
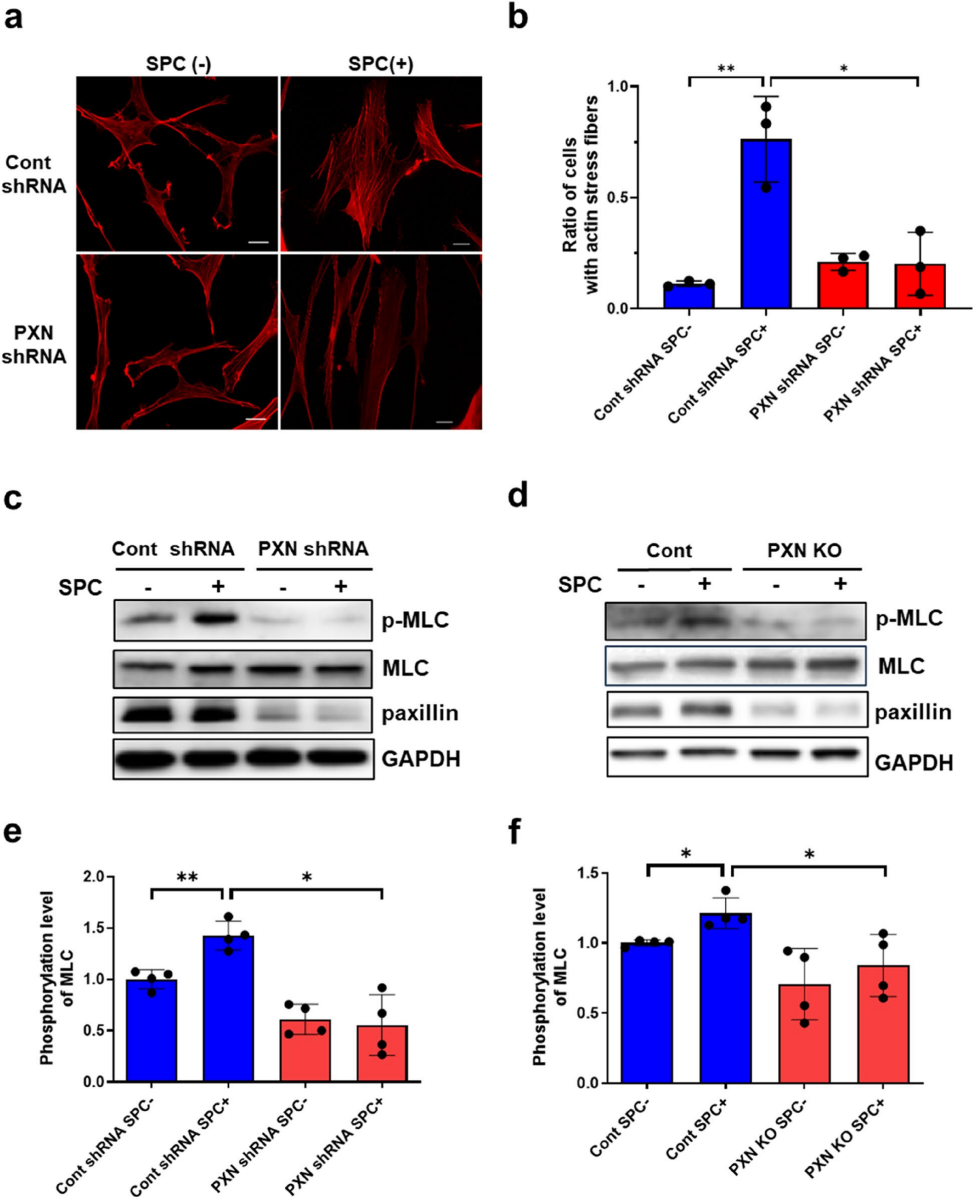
Paxillin knockdown inhibits SPC-induced actin stress fiber formation and myosin light chain phosphorylation. **a**, Immunofluorescence staining of the SPC-induced actin stress fiber formation in control and paxillin knockdown human CASMCs. Scale bar = 20 µm. **b**, Statistical analysis of the SPC-induced actin stress fiber formation in control and paxillin knockdown human CASMCs. Data were obtained from three independent experiments, and at least 44 cells were counted. **c** and **d**, Western blot showing the myosin light chain (MLC) phosphorylation in paxillin knockdown human CASMCs (c) and paxillin SMKO mice (d). **e** and **f**, Statistical analysis of MLC phosphorylation in paxillin knockdown human CASMCs (e, *n* = 4) and paxillin SMKO mice (f, *n* = 4). *p<0.05; **p<0.01. Cont: control mice; PXN KO: paxillin SMKO mice

Smooth muscle contraction requires myosin activation and actin cytoskeleton remodeling [33, 37–41]. Rho-kinase activation has two effects: (1) Directly phosphorylating MLC at S19 [42, 43] or phosphorylating MYPT1, which is a subunit of myosin light chain phosphatase (MLCP), inactivates MLCP and increases phosphorylation of MLC; (2) Promoting the formation of actin stress fibers [44, 45]. Since paxillin is an upstream molecule of Rho-kinase, it is hypothesized that paxillin deficiency could attenuate MLC phosphorylation and actin stress fiber formation. To investigate this, we first observed the effect of paxillin on SPC-induced actin stress fiber formation in human CASMCs. Compared to control cells, the SPC-induced actin stress fiber formation was inhibited in paxillin knockdown cells (Fig. 6a, b). Next, we examined MLC phosphorylation in control cells, which showed that SPC stimulation induced MLC phosphorylation. However, the SPC-induced MLC phosphorylation was significantly decreased in paxillin knockdown cells (Fig. 6c, e). Similarly, the SPC-induced MLC phosphorylation was significantly reduced in paxillin SMKO mice compared to control mice (Fig. 6d, f). These results suggest that paxillin regulates two contractile factors, actin cytoskeleton remodeling and myosin activation, by regulating the activation of Rho-kinase, which in turn regulates the SPC-induced contraction of VSM.

## Discussion

In this study, we generated paxillin knockout mice targeting smooth muscle for the first time and reported the following key findings: (1) paxillin deficiency attenuates abnormal contraction of VSM induced by SPC but not K^+^ depolarization-induced or NA-induced contraction; (2) paxillin acts as an intermediate molecule between Fyn and Rho-kinase to participate in the SPC-induced abnormal contraction; (3) paxillin deficiency inhibits the SPC-induced actin stress fiber formation; (4) paxillin deficiency inhibits the SPC-induced MLC phosphorylation; (5) in human CASMCs, paxillin knockdown also inhibits the SPC-induced contraction, Rho-kinase activation, and MLC phosphorylation. These key findings indicate that paxillin controls Rho-kinase activation and participates in the SPC-induced abnormal contraction of VSM. The revealed mechanism will provide new insights into cardiovascular and cerebrovascular diseases caused by abnormal contraction of VSM.

In the present study, we discovered a novel function of paxillin, which is involved in regulating abnormal contraction of VSM. While previous research has extensively studied the role of paxillin in cell migration, there is limited information regarding its effect on muscle contraction. Several studies have reported that paxillin is necessary for active tension development during Ca^2+^-dependent contraction of canine tracheal smooth muscle [30, 46]. However, to our knowledge, no research has yet investigated the role of paxillin in abnormal contraction of VSM, which occurs in a Ca^2+^-independent manner. So far, no paxillin inhibitor is currently available for in vitro and in vivo studies. Therefore, we employed a loss-of-function approach to investigate the role of paxillin in abnormal contraction of VSM induced by SPC. Initially, we generated paxillin knockdown human CASMCs through the use of paxillin shRNA and observed a significant inhibition of SPC-induced contraction in these cells (Fig. 1c, d). We then sought to investigate the role of paxillin in animal vascular tissue. However, mice with a whole-body knockout of paxillin displayed embryonic lethality at E9.5 due to defects in the amnion and allantois, as well as impaired growth, abnormal heart development, and impaired somite development [47]. As a result, we utilized a tamoxifen-inducible Cre-LoxP system to generate temporally selectable and spatially specific paxillin SMKO mice in this study. In this system, Cre recombinase is placed downstream of the smooth muscle myosin heavy chain (SMMHC) promoter, ensuring smooth muscle cell (SMC)-specific expression of Cre recombinase [48]. Because Cre is fused to a mutated human estrogen receptor (ER^T2^), its activity can be induced by tamoxifen [49]. When mice are administrated with tamoxifen, Cre is expressed and then enters into the nucleus. Cre catalyzes site-specific recombination events between loxP sites, thus smooth muscle tissue-specific paxillin knockout mice are obtained [34]. On the other hand, the expression of paxillin in cardiomyocytes and skeletal muscle cells are not affected (Fig. 2 and Additional file 3: Figure S3). Paxillin deficiency in VSM of mice also resulted in a decreased contraction induced by SPC (at 3 µM and 10 µM, Fig. 4), further supporting the critical role of paxillin in the abnormal VSM contraction. However, at a higher dose of SPC (30 µM), no significant difference in vasoconstriction was observed between control and paxillin SMKO mice (Additional file 4: Figure S4). It should be noted that the concentration of SPC in human plasma and serum is typically reported to be approximately 50 nM and 130 nM, respectively [50]. Although the exact concentration of SPC in mice plasma is unknown, the concentration of 30 µM SPC used in this experiment may significantly exceed physiological levels, potentially inducing vasoconstriction in paxillin-deficient mice.

Our recent study showed that paxillin binds to the active Fyn, but not the inactive Fyn [32], indicating that Fyn is activated by SPC and subsequently combines with paxillin. Here, we further showed that paxillin deficiency did not impact the Fyn activation (Fig. 5a, b), suggesting that paxillin, as a downstream molecule of active Fyn, is involved in the SPC-induced contraction of VSM. In addition, paxillin deficiency in human CASMCs or in mice inhibited the Rho-kinase activation, indicating that paxillin, as an upstream molecule of Rho-kinase, regulates the Rho-kinase-mediated contraction of VSM. Thus, paxillin acts as an intermediate regulatory molecule between Fyn and Rho-kinase to mediate abnormal contraction of VSM induced by SPC.

Smooth muscle contraction results from the interaction between myosin filament and actin filament. Actin filament polymerization and myosin activation are two parallel cellular processes that are essential for regulating smooth muscle contraction [39]. There are two types of contractions in vascular smooth muscle: Ca^2+^-dependent and Ca^2+^-independent contractions. Ca^2+^-dependent contraction, including high K^+^ depolarization-induced or NA-induced contraction, maintains physiological contraction through the Ca^2+^/CaM-MLCK signaling pathway. On the other hand, Ca^2+^-independent contraction induced by SPC causes pathological vasospasm via the Fyn/Rho-kinase signaling pathway. Our experiments on paxillin SMKO mice showed that the high K^+^ depolarization-induced or NA-induced Ca^2+^-dependent contractions were not inhibited, indicating that paxillin may not play an important role in physiological, Ca^2+^-dependent contraction (Fig. 3b,c). However, we observed that paxillin deficiency reduced SPC-induced Ca^2+^-independent contraction (Fig. 4). Furthermore, our results have shown that paxillin plays a dual regulatory role, which is involved in regulation of actin polymerization and myosin light chain phosphorylation by controlling of Rho-kinase activation. Rho-kinase activation not only promotes actin polymerization [45] but also increases MLC phosphorylation by inhibiting MLC phosphatase [51] or by directly phosphorylating MLC [43]. Paxillin deletion inhibited the SPC-induced Rho-kinase activation, suggesting that paxillin could be involved in two processes of abnormal VSM contraction, actin polymerization (Fig. 6a, b) and myosin activity (Fig. 6c-f), through Rho-kinase activation.

## Conclusions

**Fig. 7:**
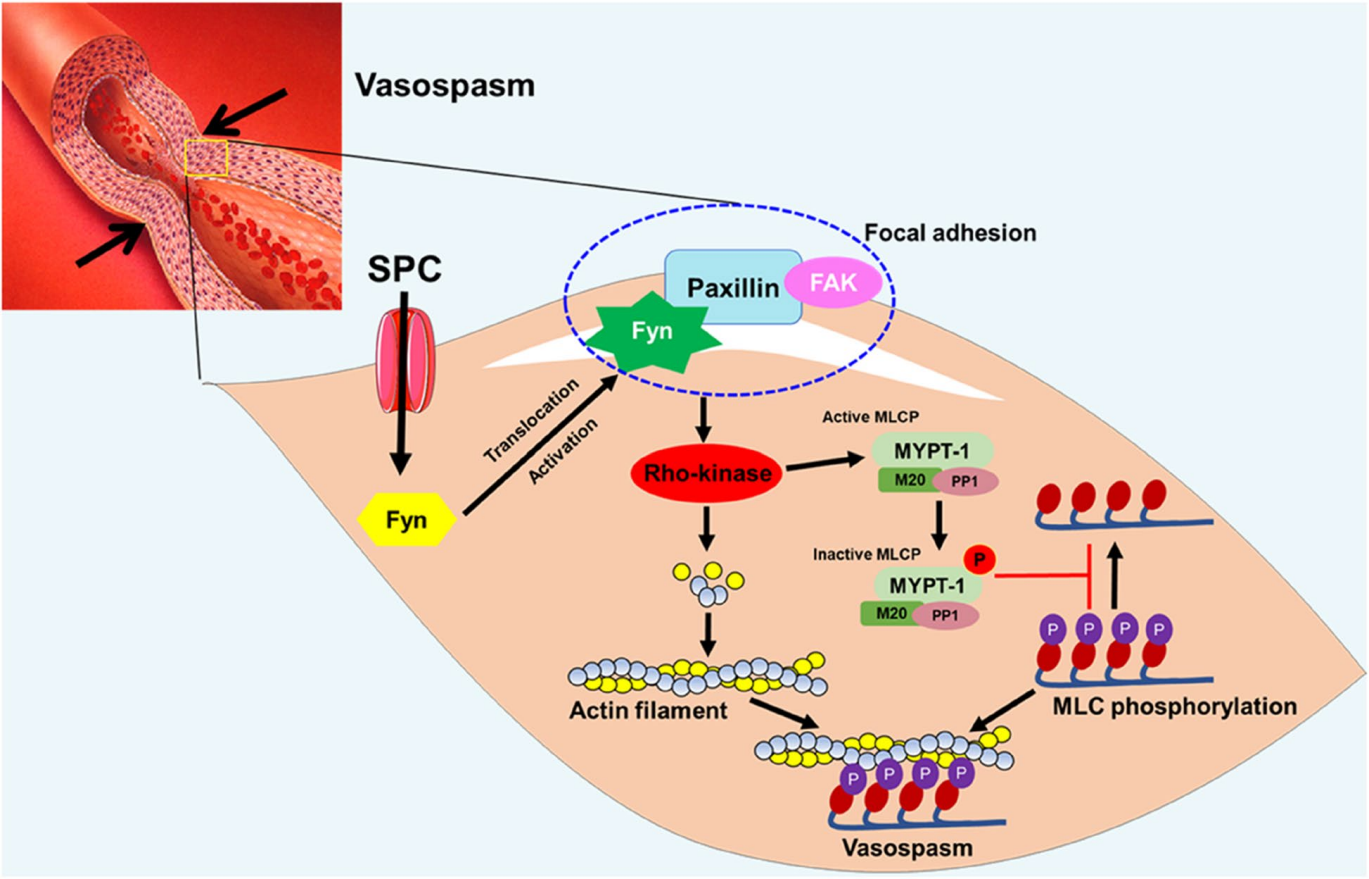
A schematic diagram showing that paxillin participates in the SPC-induced vascular smooth muscle contraction mediated by the Fyn/paxillin/Rho-kinase pathway. When stimuli factor SPC acts on vascular smooth muscle cells, Fyn is activated and translocated to the focal adhesion to bind to paxillin, resulting in Rho-kinase activation, which increases actin stress fiber formation and phosphorylation of MLC, triggering vascular smooth muscle abnormal contraction (vasospasm)

Collectively, we have provided evidence for the first time that paxillin, a novel regulatory molecule, participates in the abnormal contraction of VSM mediated by the SPC/Fyn/paxillin/Rho-kinase pathway (Fig. 7). Our findings suggest that targeting paxillin could be a promising therapeutic strategy for treating cardiovascular and cerebrovascular diseases caused by abnormal VSM contraction.

### Supplementary information

**Additional file 1: Figure S1.** Targeting vector design of smooth muscle-specific paxillin knock out mice.

**Additional file 2: Figure S2.** Establishment of paxillin SMMHC-CreER^T2^ transgenic mice.

**Additional file 3: Figure S3.** The expression of paxillin in heart muscle, skeletal muscle, and vascular smooth muscle from paxillin SMKO mice.

**Additional file 4: Figure S4.** 30 μM SPC-induced contraction in control and paxillin SMKO mice.

**Additional file 5: Video S1.** Time-lapse video of the SPC-induced contraction in control shRNA human CASMCs.

**Additional file 6: Video S2.** Time-lapse video of the SPC-induced contraction in paxillin shRNA human CASMCs.

**Additional file 7.** Supplementary uncropped images of western blot.

## Data Availability

Data can be made available upon request of the corresponding author.

## Abbreviations

SPC: Sphingosylphosphorylcholine
CASMCs: Coronary artery smooth muscle cells
VSM: Vascular smooth muscle
CaM: Calmodulin
MLCK: Myosin light chain kinase
CA-Fyn: Constitutively active Fyn
FAK: Focal adhesion kinase
MYPT1: Myosin phosphatase targeting subunit 1
SMKO: Smooth muscle knockout
NA: Noradrenaline
MLC: Myosin light chain
MLCP: Myosin light chain phosphatase
