# Supplementary figures and images for: Paxillin participates in the sphingosylphosphorylcholine-induced abnormal contraction of vascular smooth muscle by regulating Rho-kinase activation
Source: Cell Commun Signal. 2024 Jan 22;22:58. doi: 10.1186/s12964-023-01404-w (PMC10801962; doi:10.1186/s12964-023-01404-w)

# Figure S1

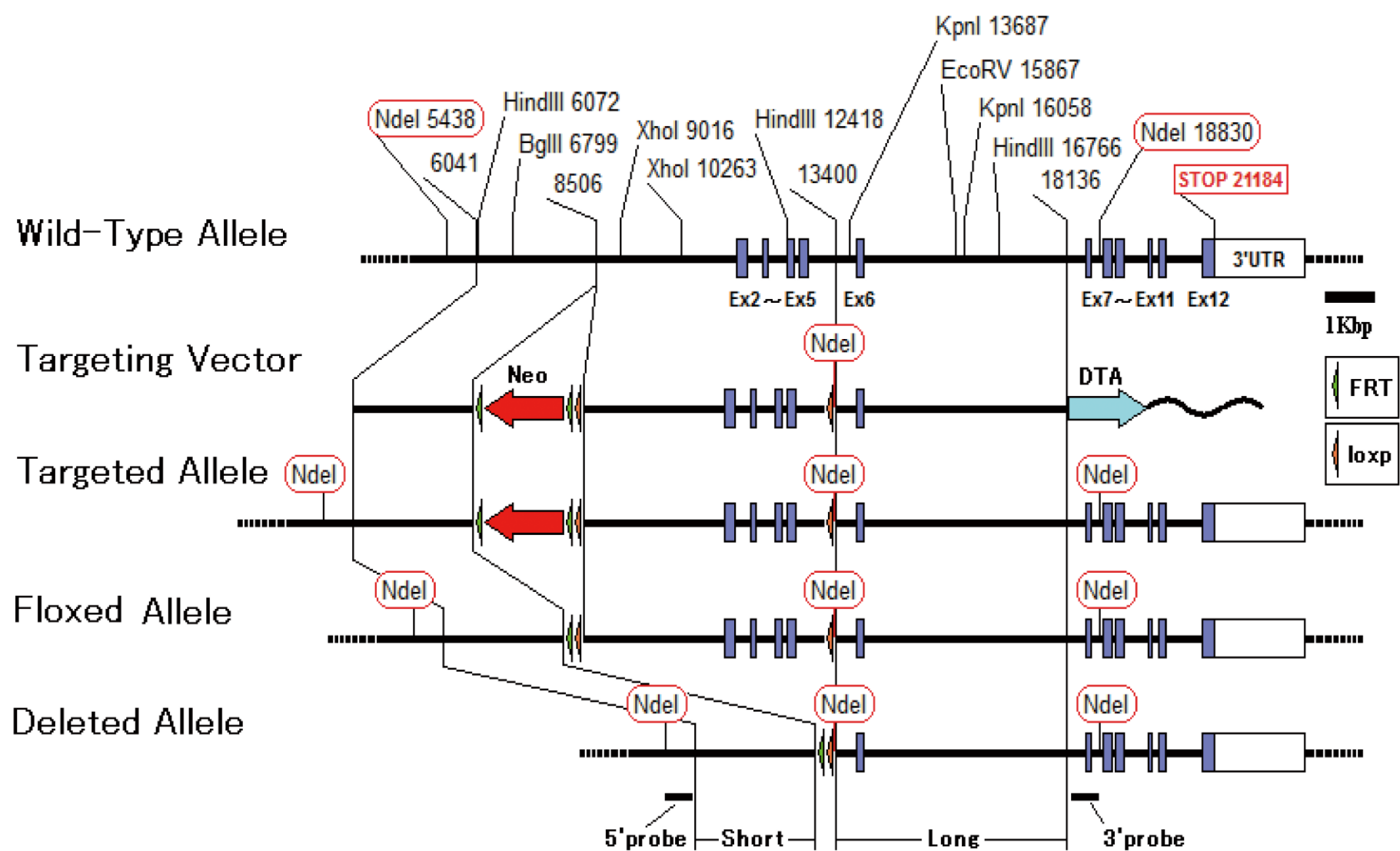

Figure S1 Targeting vector design of smooth muscle-specific paxillin knockout mice.

Supplement: Supplementary file 2 — Additional file 1: Figure S1. Targeting vector design of smooth muscle-specific paxillin knock out mice. [file 12964_2023_1404_MOESM1_ESM.pdf]
