# Supplementary material for: Paxillin participates in the sphingosylphosphorylcholine-induced abnormal contraction of vascular smooth muscle by regulating Rho-kinase activation
Source: Cell Commun Signal. 2024 Jan 22;22:58. doi: 10.1186/s12964-023-01404-w (PMC10801962; doi:10.1186/s12964-023-01404-w)
Supplement: Supplementary file 3 — Additional file 2: Figure S2. Establishment of paxillin SMMHC-CreERT2 transgenic mice. [file 12964_2023_1404_MOESM2_ESM.pdf]

Figure S2

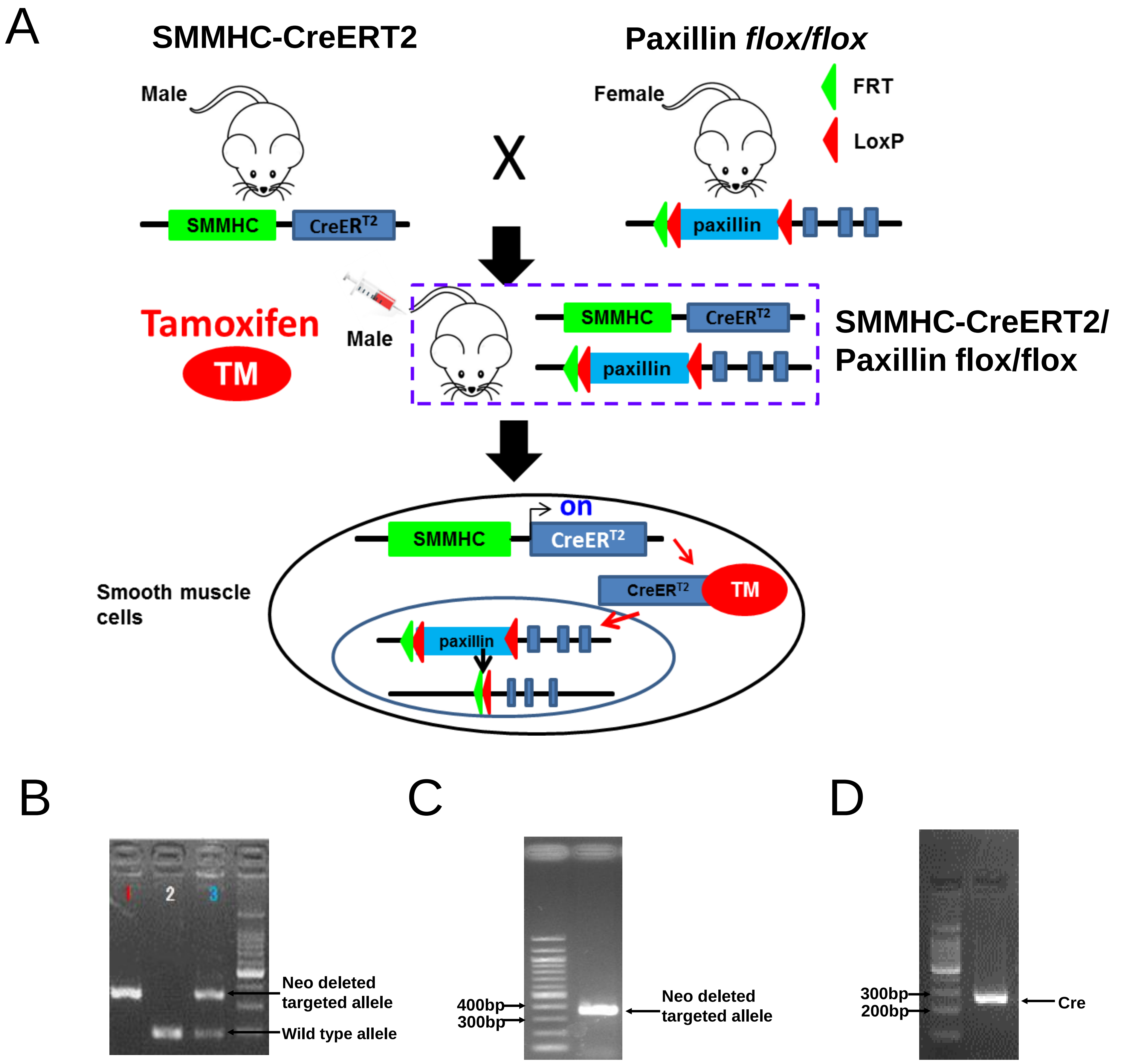

Figure S2 A, diagram showing the production of smooth muscle-specific paxillin knockout mice. B, Representative PCR analysis showing whether the neomycin sequence removes. 1: Neo-removed homo-mouse ; 2: wild type mouse; 3: Neo-removed hetero-mouse. C, Representative PCR analysis showing genotyping result of neomycin deletion from SMMHC-CreERT2/paxillin flox/flox mouse. D, Representative PCR analysis showing genotyping result of Cre expression from SMMHC-CreERT2/paxillin flox/flox mouse.
