# Supplementary material for: Paxillin participates in the sphingosylphosphorylcholine-induced abnormal contraction of vascular smooth muscle by regulating Rho-kinase activation
Source: Cell Commun Signal. 2024 Jan 22;22:58. doi: 10.1186/s12964-023-01404-w (PMC10801962; doi:10.1186/s12964-023-01404-w)
Supplement: Supplementary file 4 — Additional file 3: Figure S3. The expression of paxillin in heart muscle, skeletal muscle, and vascular smooth muscle from paxillin SMKO mice. [file 12964_2023_1404_MOESM3_ESM.pdf]

Figure S3

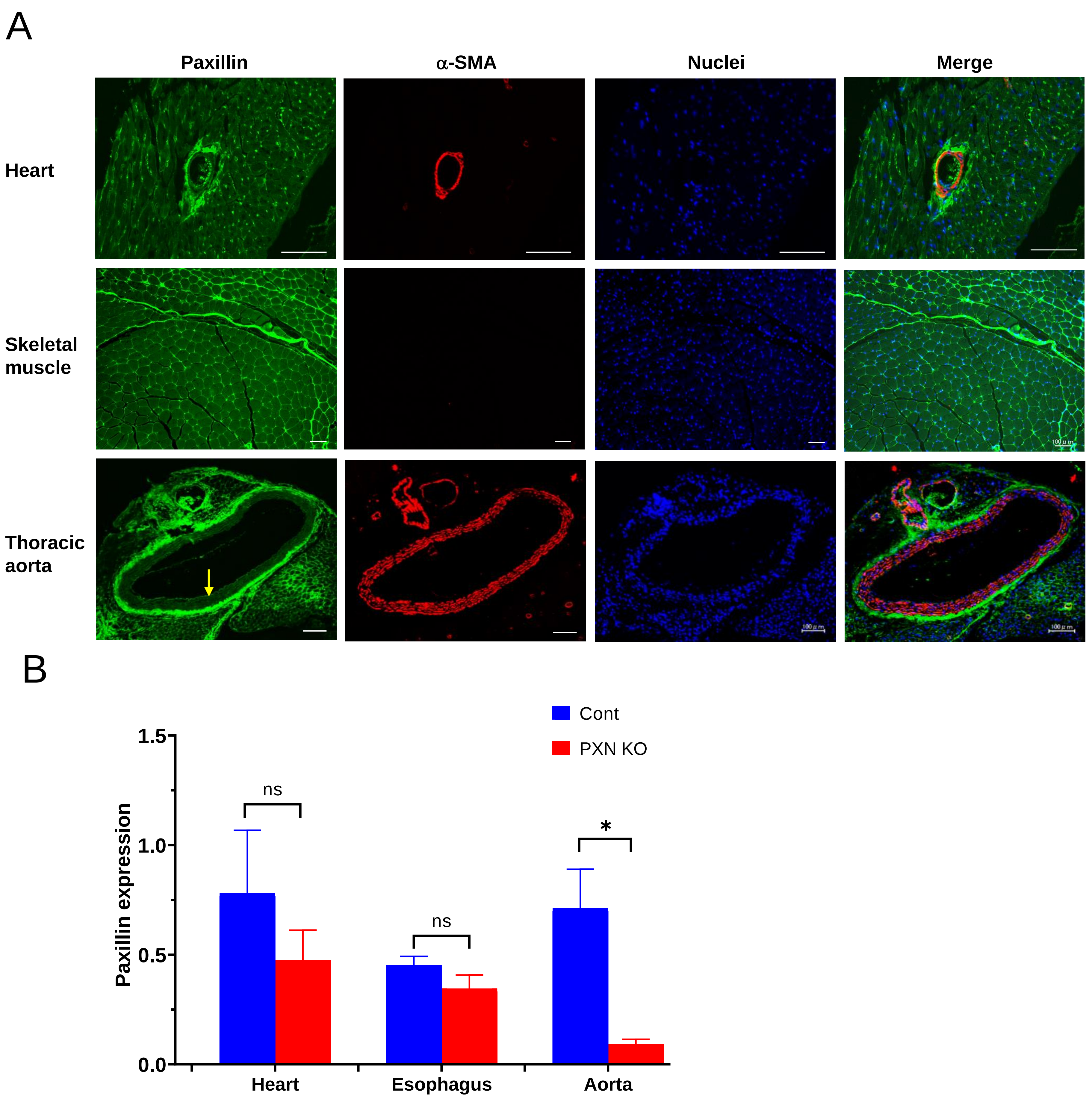

Figure S3 A, Immunofluorescence staining showing that paxillin expression in heart muscle, skeletal muscle, and vascular smooth muscle from paxillin SMKO mice. Smooth muscle α-actin (α-SMA, red) as smooth muscle-specific marker is detected in vascular smooth muscle. Paxillin (Green) is detected in the heart muscle, skeletal muscle, and non-smooth muscle around aorta but not detected in the layer of vascular smooth muscle (as shown in the yellow arrow). Scale Bar = 100 μm. B, Statistical analysis of paxillin expression in heart, esophagus, and the medial smooth muscle layer of thoracic in control and paxillin SMKO mice. *n*=3, **p*<0.05, ns: no significant. Cont: control mice; PXN KO: paxillin SMKO mice.
