# Supplementary material for: Paxillin participates in the sphingosylphosphorylcholine-induced abnormal contraction of vascular smooth muscle by regulating Rho-kinase activation
Source: Cell Commun Signal. 2024 Jan 22;22:58. doi: 10.1186/s12964-023-01404-w (PMC10801962; doi:10.1186/s12964-023-01404-w)
Supplement: Supplementary file 5 — Additional file 4: Figure S4. 30 μM SPC-induced contraction in control and paxillin SMKO mice. [file 12964_2023_1404_MOESM4_ESM.pdf]

Figure S4

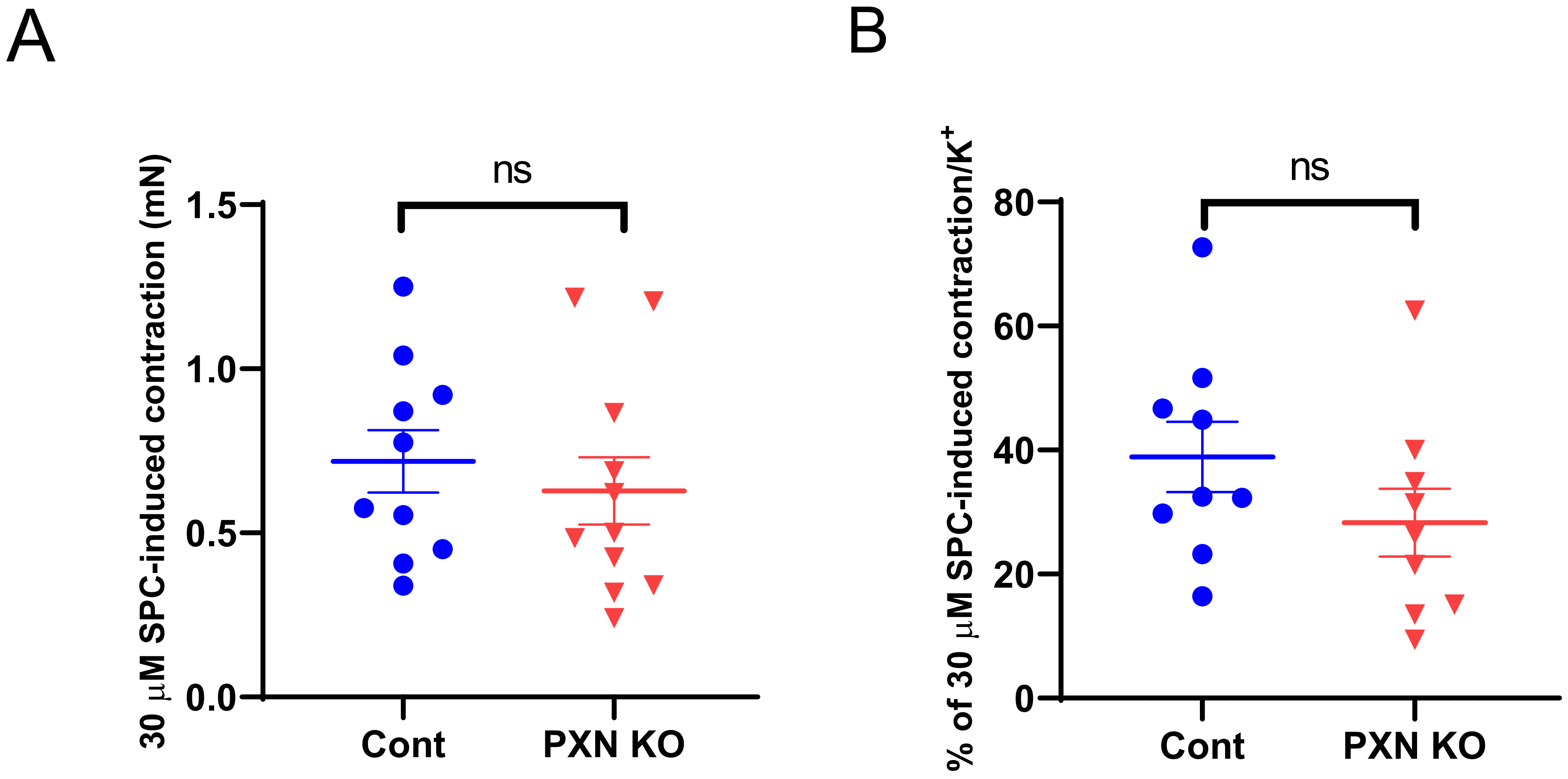

Figure S4 A, 30 μM SPC-induced contraction (mN) in control and paxillin SMKO mice. B, Ratio of 30 μM SPC-induced contraction compared to 80 mM K^+ depolarization-induced contraction in control and paxillin SMKO mice. ns, no significant. ∗p<0.05. Cont: control mice; PXN KO: paxillin SMKO mice.
