# Supplementary material for: Paxillin participates in the sphingosylphosphorylcholine-induced abnormal contraction of vascular smooth muscle by regulating Rho-kinase activation
Source: Cell Commun Signal. 2024 Jan 22;22:58. doi: 10.1186/s12964-023-01404-w (PMC10801962; doi:10.1186/s12964-023-01404-w)
Supplement: Supplementary file 8 — Additional file 7. Supplementary uncropped images of western blot. [file 12964_2023_1404_MOESM7_ESM.pdf]

Supplementary uncropped images of western blot

The parts in the red box are used in the paper.

Fig.1a

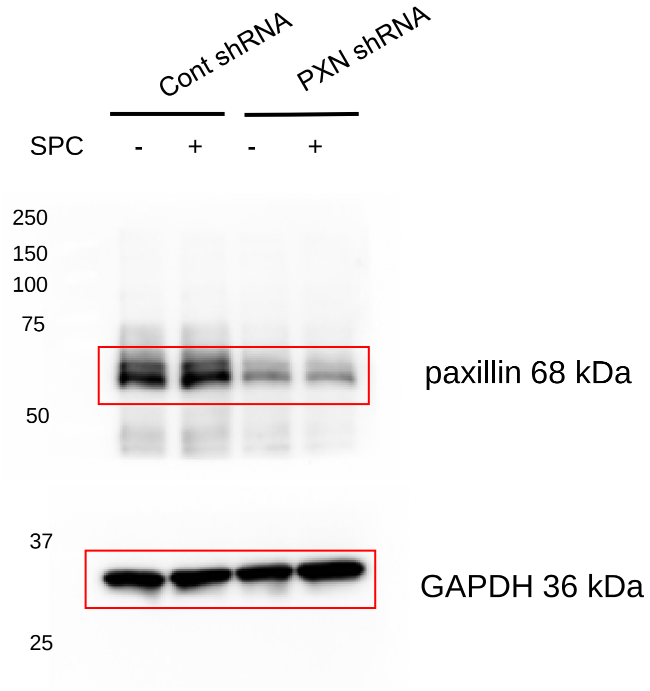

Fig.2a

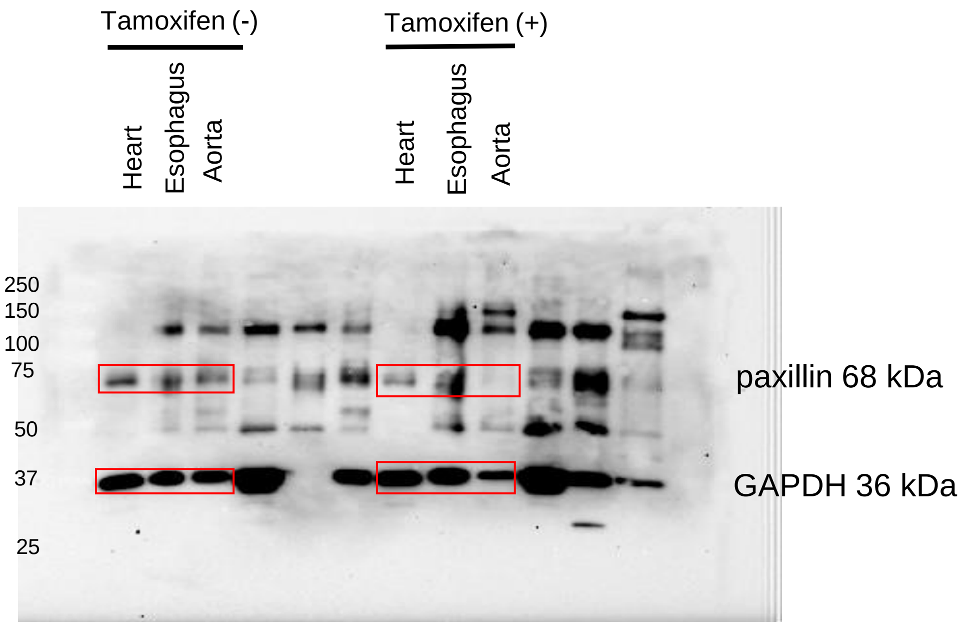

Fig.5a

IP

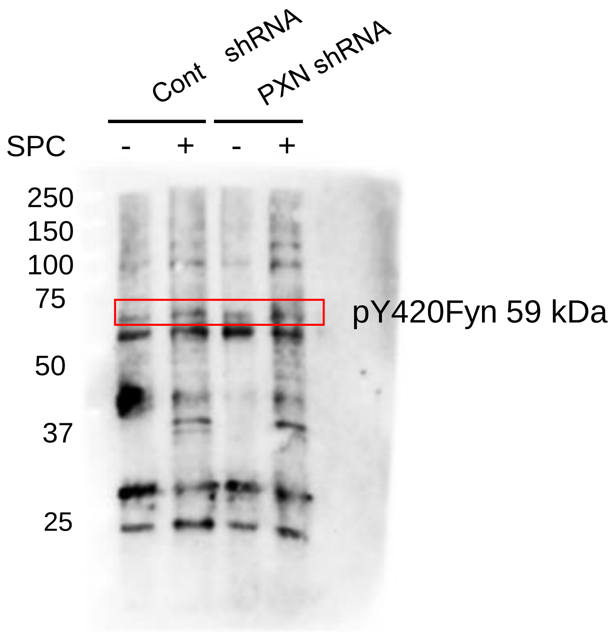

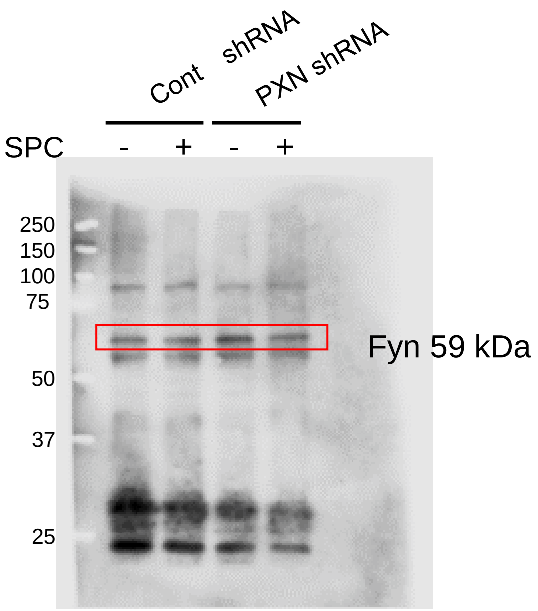

Input

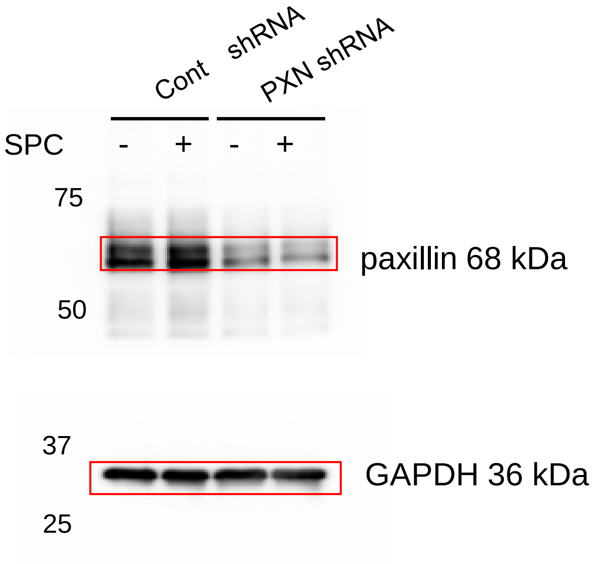

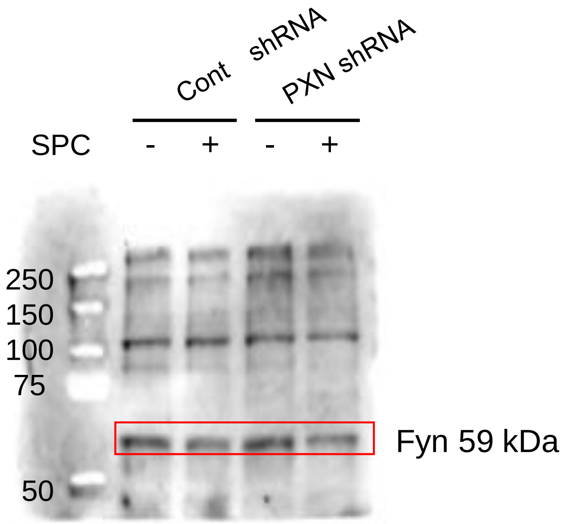

Fig.5c

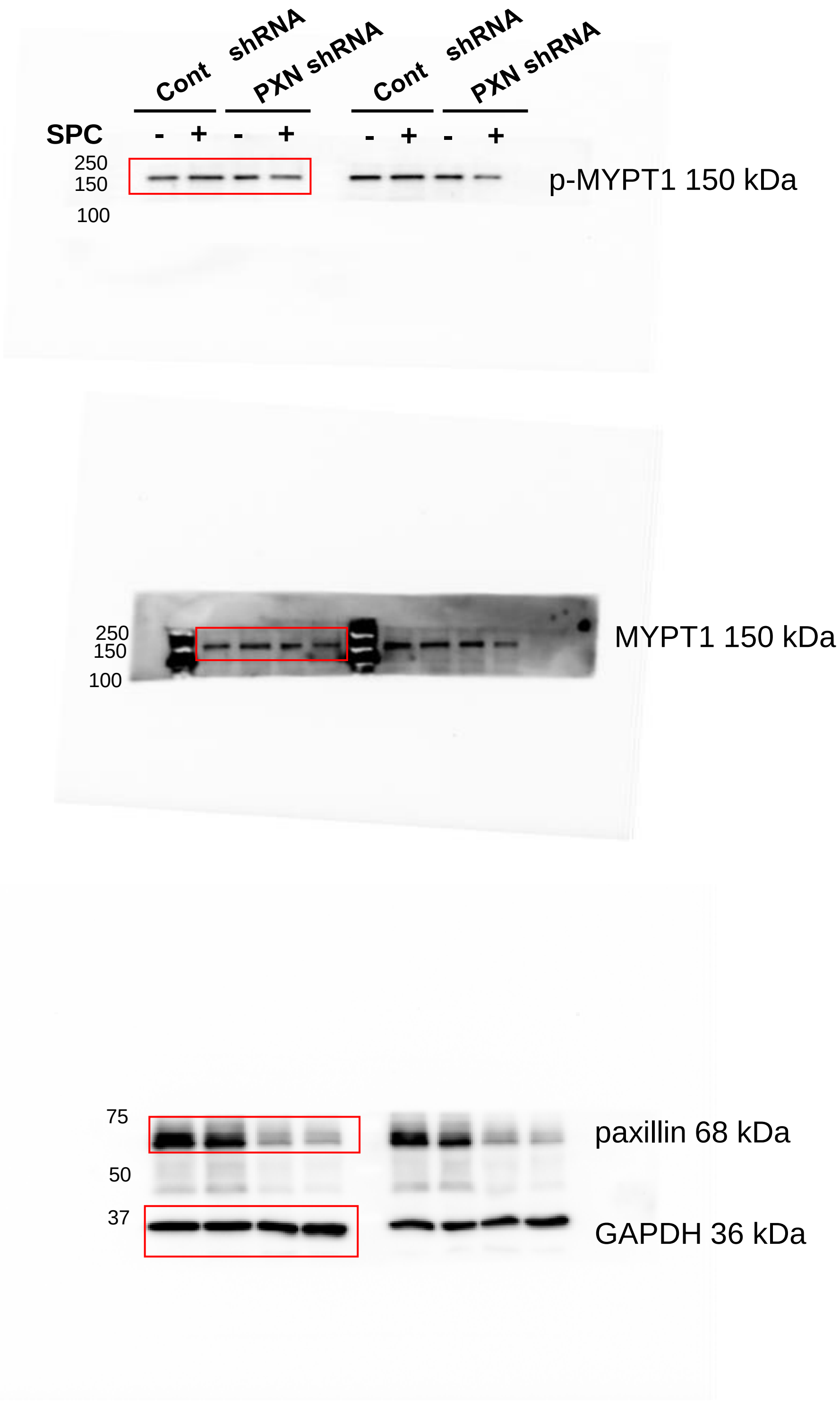
Cont shRNA
PXN shRNA
Cont shRNA
PXN shRNA
SPC
- + - + - + - +
250
150
100
p-MYPT1 150 kDa
250
150
100
MYPT1 150 kDa
75
50
37
paxillin 68 kDa
GAPDH 36 kDa

Fig.5d

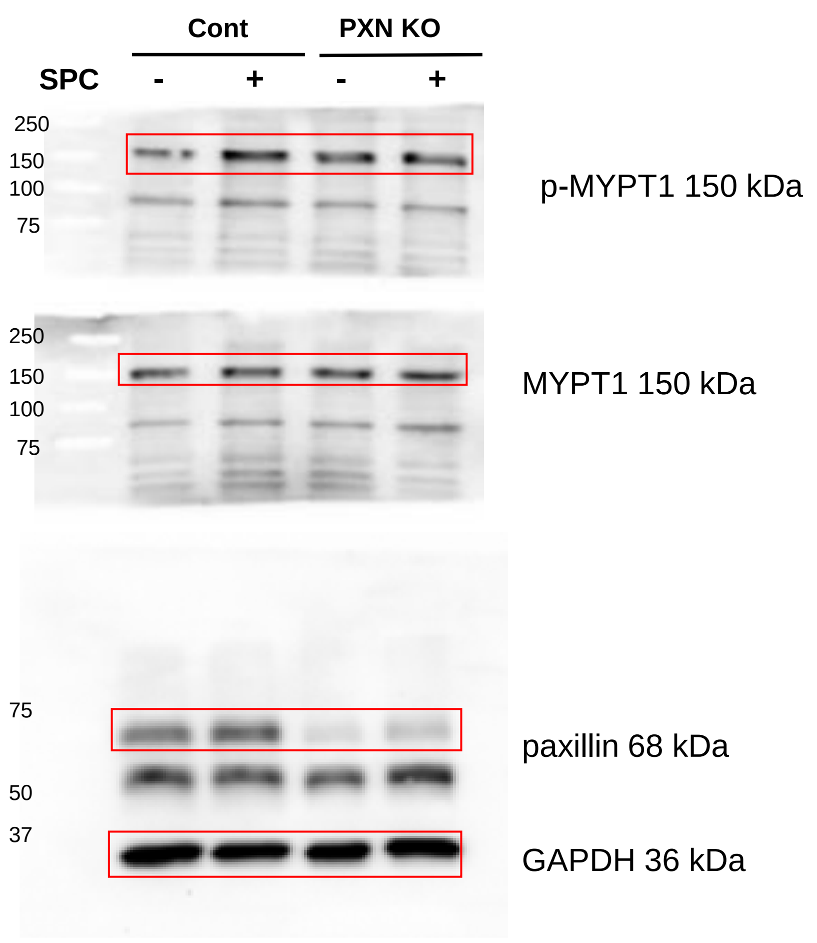

Cont
PXN KO
SPC
-
+
-
+
250
150
100
75
p-MYPT1 150 kDa
250
150
100
75
MYPT1 150 kDa
75
50
37
paxillin 68 kDa
GAPDH 36 kDa

Fig.6c

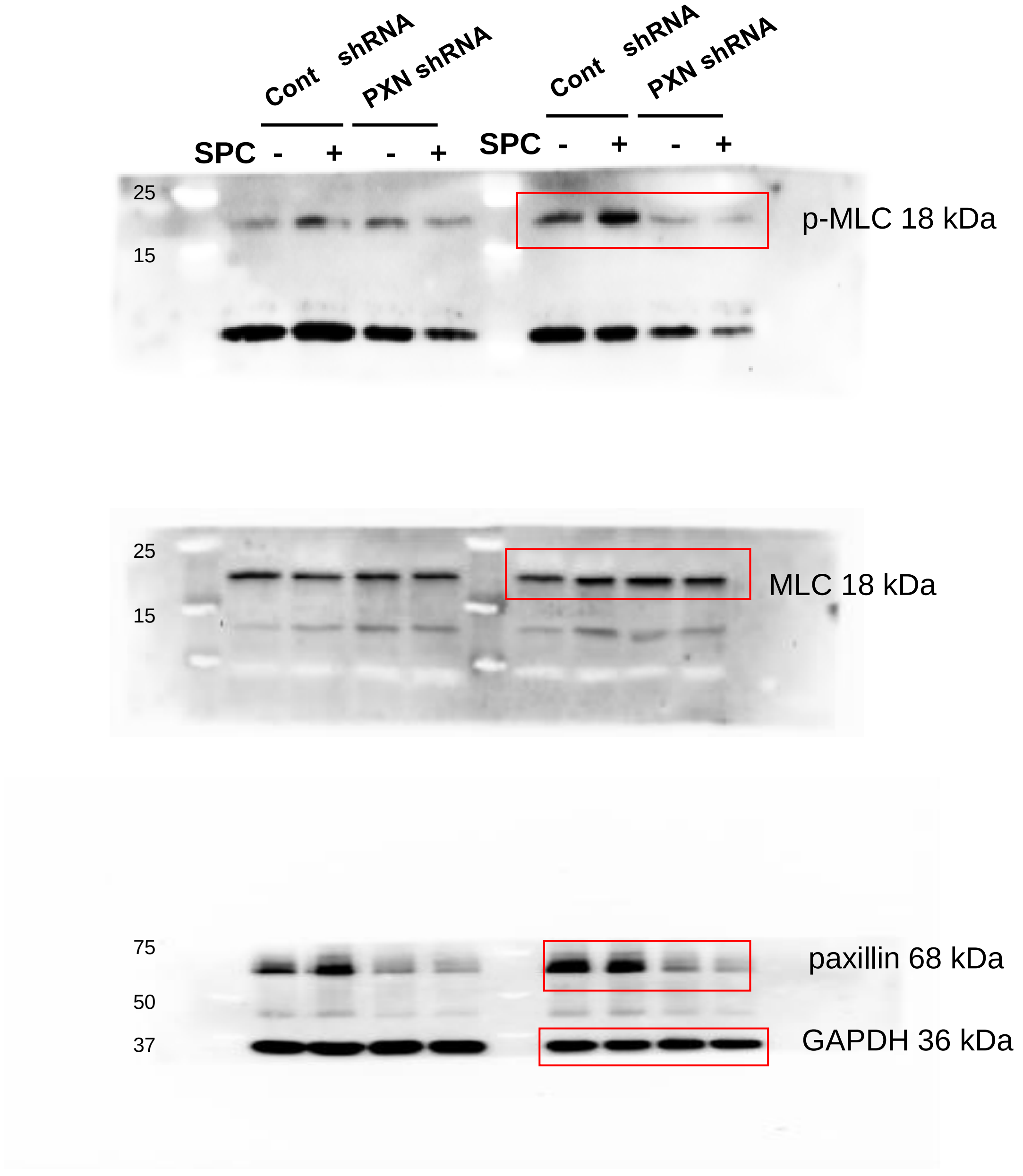

Cont shRNA
PXN shRNA
SPC - + - +
Cont shRNA
PXN shRNA
SPC - + - +
25
15
p-MLC 18 kDa
25
15
MLC 18 kDa
75
50
37
paxillin 68 kDa
GAPDH 36 kDa

Fig.6d

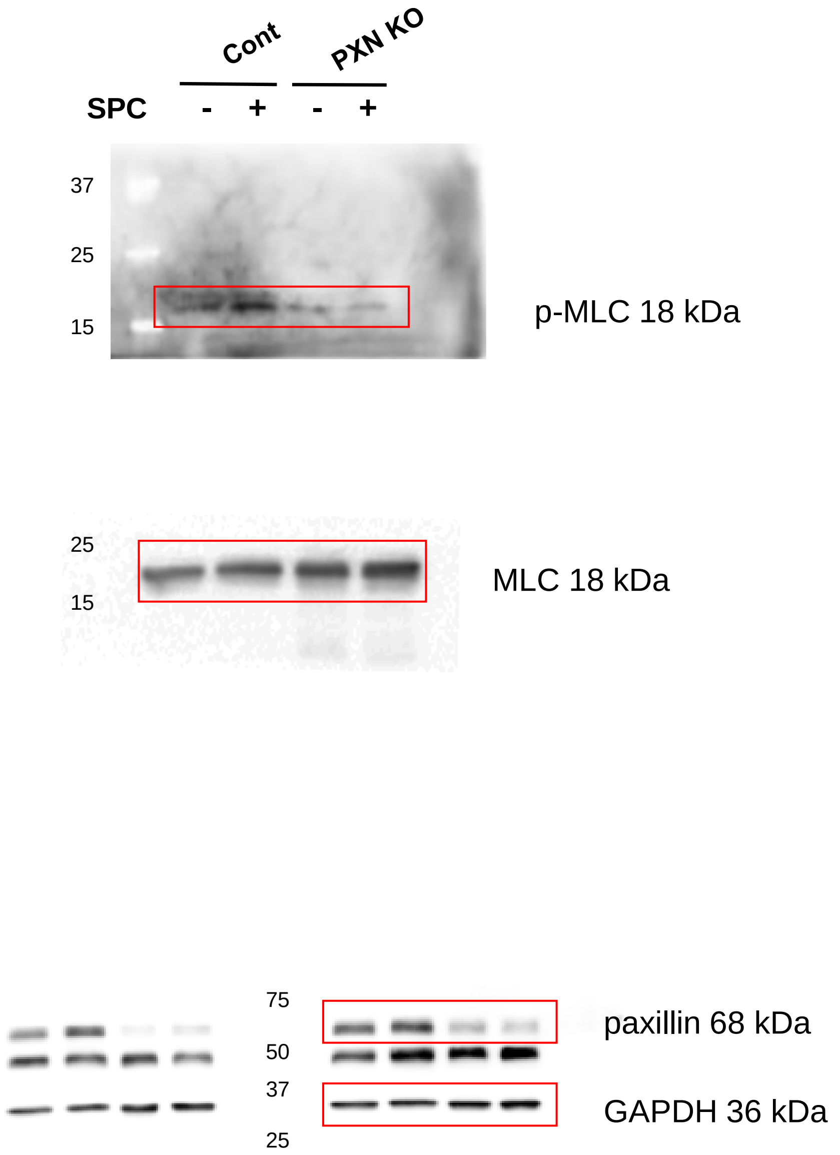

Cont
PXN KO
SPC - + - +
37
25
15
p-MLC 18 kDa
25
15
MLC 18 kDa
75
50
37
25
paxillin 68 kDa
GAPDH 36 kDa
